# 25475 Healthcare Delivery Science in LA: Addressing patient and health system priorities with cross-sector research infrastructure

**DOI:** 10.1017/cts.2021.724

**Published:** 2021-03-30

**Authors:** Amytis Towfighi, Allison Z. Orechwa, Arleen F. Brown, Moira Inkelas, Stefanie Vassar, Deborah K. Herman

**Affiliations:** 1 University of Southern California and LA County Department of Health Services; 2 Southern California Clinical and Translational Science Institute; 3 University of California Los Angeles and LA County Department of Health Services; 4 University of California Los Angeles; 5 University of California Los Angeles Clinical and Translational Science Institute

## Abstract

ABSTRACT IMPACT: Effective healthcare interventions improve access, quality of care, and health outcomes for underserved, high-disparity populations of Los Angeles county and beyond. OBJECTIVES/GOALS: We will expand our successful, Los Angeles-based public-academic partnership to develop and evaluate health system interventions aimed at improving healthcare for underserved communities, as well as develop workforce skilled in healthcare delivery science. METHODS/STUDY POPULATION: Together with the LA County Department of Health Services, the two LA-based CTSA hubs at USC and UCLA have established critical infrastructure for effective cross-sector translational research: (1) New funding mechanisms to evaluate health system interventions in county hospitals and clinics in areas of mutual interest; (2) Specialized research service cores (Safety-net Health Innovation core, Clinical Research Informatics core, and Healthcare Delivery Science core), and (3) Training and mentorship programs tailored for healthcare delivery scientists. RESULTS/ANTICIPATED RESULTS: Outcomes from the first four years of the partnership include: (1) Significant impact on health outcomes from eight funded projects, e.g., lowered A1c levels by 0.9%; (2) Successful, coordinated service to dozens of research projects, e.g., a teleretinal screening program decreased ophthalmology visit wait times from 158 to 17 days; (3) New virtual coursework in seven domains (healthcare delivery science, dissemination and implementation science, systems engineering, behavioral economics, informatics, team science, and community engagement); (4) A published ‘synergy paper’ w/ CTSA hubs in three other urban cities examining common themes of academic-public partnerships; and (5) Rapid and streamlined COVID-19 research policy setting with county leadership. DISCUSSION/SIGNIFICANCE OF FINDINGS: Our sustainable infrastructure is effectively bridging research-policy-practice gaps in Los Angeles and addressing patients’ and the health system’s priorities.

